# Effects of dietary supplementation with histidine and β-alanine on blood plasma metabolome of broiler chickens at different ages

**DOI:** 10.1371/journal.pone.0277476

**Published:** 2022-11-14

**Authors:** Julia Lackner, Vincent Hess, Achim Marx, Morteza Hosseini-Ghaffari, Helga Sauerwein

**Affiliations:** 1 Evonik Operations GmbH, Hanau-Wolfgang, Germany; 2 Institute of Animal Science, Physiology Unit, University of Bonn, Bonn, Germany; 3 Evonik Digital GmbH, Essen, Germany; Tokat Gaziosmanpasa Universitesi, TURKEY

## Abstract

Histidine is an essential amino acid for broiler chickens and a precursor for the dipeptides carnosine and anserine, but little information is available about its metabolism in modern, fast-growing broilers. We used untargeted metabolomics to investigate the metabolic changes caused by the use of different standardized ileal digestible His:Lys ratios in broiler diets with and without β-alanine supplementation. A total of 2204 broilers were randomly divided into 96 pens of 23 birds each. The pens were divided into 16 blocks, each containing one pen for all six feeding groups (total of 16 pens per group). These feeding groups were fed three different His:Lys ratios (0.44, 0.54, and 0.64, respectively) without and with a combination of 0.5% β-alanine supplementation. Five randomly selected chickens of one single randomly selected pen per feeding group were slaughtered on day 35 or 54, blood was collected from the neck vessel, and plasma was used for untargeted metabolomic analysis. Here we show that up to 56.0% of all metabolites analyzed were altered by age, whereas only 1.8% of metabolites were affected by the His:Lys ratio in the diet, and 1.5% by β-alanine supplementation. Two-factor analysis and metabolic pathway analysis showed no interaction between the His:Lys ratio and β-alanine supplementation. The effect of the His:Lys ratio in the diet was limited to histidine metabolism with a greater change in formiminoglutamate concentration. Supplementation of β-alanine showed changes in metabolites of several metabolic pathways; increased concentrations of 3-aminoisobutyrate showed the only direct relationship to β-alanine metabolism. The supplementation of β-alanine indicated few effects on histidine metabolism. These results suggest that the supplements used had limited effects or interactions on both His and β-alanine metabolism. In contrast, the birds’ age has the strongest influence on the metabolome.

## Introduction

Histidine (**His**) is an essential amino acid for many species, including poultry [1, 2]. With its unique aromatic imidazole side chain, His can function both as a proton donor and acceptor under physiological conditions and is important for the structure of proteins and for the catalytic activity of enzymes [3–7]. His and its related molecules can act as metal ion chelators, pH buffers, antioxidants, or extracellular messengers [3, 6, 8–11], which makes them important molecules under physiological conditions. The His metabolism consists of several known metabolic pathways: the main catabolism to glutamate, the catabolism through the intermediates imidazole pyruvate and imidazole lactate and the decarboxylation of His to form histamine [3, 6] which acts as a signaling molecule in many tissues and metabolic pathways [12]. Other metabolites include acetylated and methylated His forms. In vertebrates, His-related dipeptides can also be found in relevant concentrations in skeletal and cardiac muscle tissue and in some parts of the brain [9]. These dipeptides have important functions and can act as intracellular buffers, metal-ion chelators, and antioxidants [9]. In chickens, carnosine, synthesized from His and the non-essential amino acid β-alanine (**βA**), as well as its methylated form anserine, are the dominating His-related dipeptides [9, 13]. The second precursor amino acid βA is formed as an intermediate of the metabolic pathways of aspartate and uracil [14, 15].

Dietary recommendations for the ratio of His to lysine (**Lys**) vary from 0.31 to 0.41 in broiler chickens [16–20]. In commercial production, His is not considered a limiting factor, and thus receives little attention in feed formulation. Nevertheless, carnosine depletion has been described in birds suffering from breast muscle myopathies such as white striping, woody breast, and spaghetti meat [21, 22]. Therefore, higher feeding levels of His could help to increase the concentration of this important dipeptide and counteract the development of breast myopathies. However, less is known about the overall metabolism of broilers fed higher His concentrations and the effect of βA supplementation on metabolism. The metabolomic study described in this paper is based on a feeding trial in which the effects of three different standardized ileal digestible His:Lys ratios in the diets of fast-growing broiler chickens with or without βA supplementation were tested at different ages [23]. This previous study showed less influence of the His:Lys ratio or βA supplementation on meat quality parameters, but an increase in carnosine concentration with a higher His:Lys ratio in feed, but without βA supplementation having an effect on the concentration of this dipeptide. In addition, with generally little influence on the performance of the animals, it was shown that His feeding could lead to an increase in mortality in older animals. The current study aimed at complementing the previous study by identifying the main metabolites impacted by His or βA supplementation and the possible interaction of both. Additionally, the impact of birds’ age on metabolism, especially His metabolism, should be further studied at different ages using an untargeted metabolomics approach. It was found that age had a major impact on broiler metabolism. The His:Lys ratio in feed mainly affected the targeted His metabolism, while a supplementation of βA had only marginal effects on a few different metabolites. A supplementation of βA, as opposed to His, had no effect on carnosine metabolism. In addition, it was found that with higher His:Lys rations in feed the concentrations of formiminoglutamate in blood plasma of broiler chicken was increased, which is a marker for folate or cobalamin deficiency [24, 25].

## Materials and methods

### Study design and sampling

**Fig 1:**
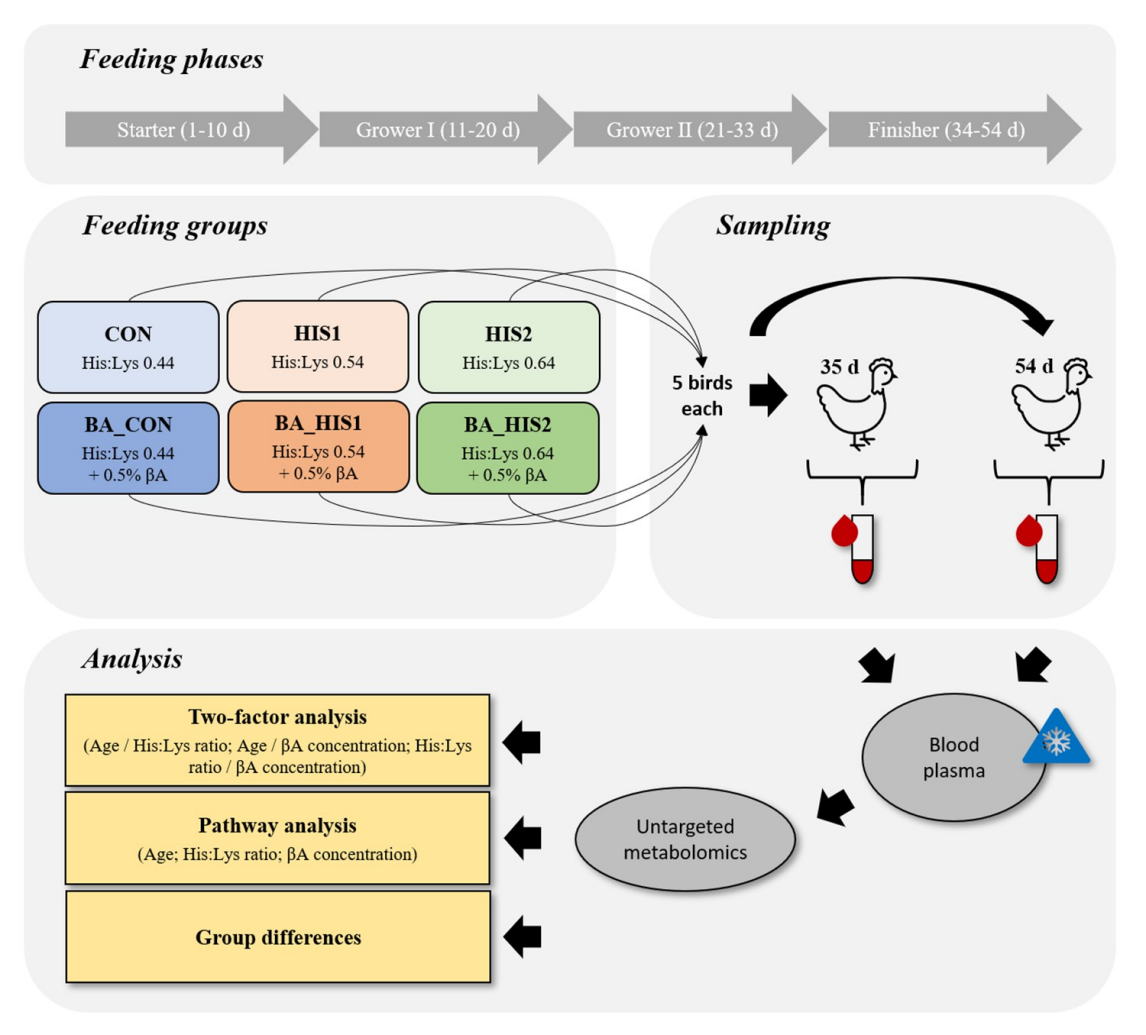
Study design. Abbreviations: His:Lys, standardized ileal digestible histidine:lysine ratio; βA, β-alanine; CON, group fed with a His:Lys ratio of 0.44; HIS1, group fed with a His:Lys ratio of 0.54; HIS2, group fed with a His:Lys ratio of 0.64; BA_CON, group fed with a His:Lys ratio of 0.44 + supplementation of 0.5% β-alanine; BA_HIS1, group fed with a His:Lys ratio of 0.54 + supplementation of 0.5% β-alanine; BA_HIS2, group fed with a His:Lys ratio of 0.64 + supplementation of 0.5% β-alanine.

The animals investigated in this study were kept and treated as for good farming practice but were not exposed to specific measures that could imply pain, suffering or damage. All evaluations were performed on dead animals and the killing of animals for collecting samples is not defined as animal experiment according to the German animal welfare act (TierSchG). The study followed animal welfare regulations for commercial fattening and the animal care legislation of the German animal welfare act including the EU Directive 2010/63/EU. An overview of the study design is given in Fig 1. For the present metabolomic study, plasma samples collected in a feeding trial in which the effects of standardized ileal digestible amino acid His:Lys ratios of 0.44 (**CON**), 0.54 (**HIS1**), and 0.64 (**HIS2**) combined with or without a supplementation of 0.5% total βA (**BA_CON; BA_HIS1; BA_HIS2**) on performance, His-related dipeptide content, and meat quality were tested. This entire study, including the composition of the basal diet for each feeding phase and the supplemented His and βA concentrations relative to the basal diet, is described in detail in a previous publication [23]. Briefly, the study was conducted with 2,208 Ross 308 broiler chickens randomly allocated in 96 pens with 23 birds per pen. Each adapted feeding, of the six feeding groups, was administered to the birds in a randomized block design with 16 replicates (368 birds per group). The adapted feedings of different feeding groups were given over the whole fattening period in all feeding phases (Starter 1–10 d, Grower I 11–20 d, Grower II 21–33 d, Finisher 34–54 d). At 35 and 54 days of age, five different birds from a randomly selected pen per feeding group were randomly selected for blood sampling. The same birds were also used for performance analysis and targeted analysis of molecules in the breast muscle, but not for further studies [23]. To take the samples, birds were stunned by hitting the head, which caused a lack of consciousness, and then killed in a slaughter funnel by severing the neck vessel. Approximately 7 mL of blood were immediately collected from the neck vessel during bleeding and placed in K_3_ EDTA-containing vacutainers (polyethylene terephthalate, Greiner Bio-one, Frickenhausen, Germany) to prevent blood clotting. The blood was gently swirled by hand and briefly placed in a roller mixer. Samples were then centrifuged (1500 *g*, 4°C, 10 min) and 200 μL of plasma were transferred to a pre-chilled 2 mL cryovial (polypropylene, Simport Scientific, Saint-Mathieu-de-Beloeil, QC, Canada). The tubes were immediately cooled in liquid nitrogen, stored at -80°C, and further used for the metabolomics analysis.

### Metabolomics analysis

For the metabolomics analyses, the samples were transferred on dry ice to Metabolon Inc. (Morrisville, NC, USA) where four methods were performed on their HD4 platform: Ultra-High-Performance Liquid Chromatography coupled with Tandem Mass Spectrometry (UPLC-MS/MS), whereby two methods applied reverse-phase UPLC-MS/MS with electrospray ionization (ESI) in positive ion mode, one method used ESI in negative ion mode, and one method used hydrophobic interaction liquid chromatography UPLC-MS/MS with ESI in negative ion mode. Metabolites were identified using the Metabolon hardware and software. All metabolites were quantified as area-under-the-curve detector ion counts and normalized with respect to the raw area counts. For standardization of the measured values internal standards of Metabolon were used.

### Data analysis

For the analysis, which takes more than one day, the original values were normalized in terms of raw area counts. For further statistical analysis all data were rescaled by the median of all 60 samples for a given molecule. Missing values were imputed with the observed minimum value for each detected metabolite after rescaling (4.8% of the overall values). A statistical heat map for age differences per feeding group (35 d and 54 d) and single group comparisons at each slaughter age was provided by Metabolon Inc. The heat map is based on the statistical differences of the respective group comparisons using pairwise comparison based on ANOVA contrasts on log-transformed data. The MetaboAnalyst 5.0 web tool (Wishart Research Group, University of Alberta, AB, Canada) was used for further analysis. The method used for two-factor analysis and pathway analysis was previously reported [26, 27]. Previously normalized and rescaled data were used for the analysis. Using MetaboAnalyst 5.0, metabolite data were transformed using the generalized log transformation. No additional filtering or normalization of the data was performed by the software. For the two-factor analysis, the study design of ‘two-factor independent samples’ was set, and two-way type I ANOVA analysis was performed to identify differences and interactions between the factors His:Lys ratio/ βA supplementation and His:Lys ratio/slaughter age in the diet and βA supplementation/slaughter age. False discovery rates (FDR) were set for the multiple test corrections with an adjusted p-value cutoff at 5%. For analyzing the metabolic pathways affected by His:Lys ratio in the diet, βA supplementation, or slaughter age as a factor, MetaboAnalyst 5.0 (Wishart Research Group, University of Alberta, Canada) was used based on the Kyoto Encyclopedia of Genes and Genomes database (KEGG, Kanehisa Laboratories, Institute for Chemical Research, Kyoto, Japan, https://www.genome.jp/kegg/). For metabolite identification, the ID numbers from the Human Metabolon Database (HMDB) were used because most metabolites were identified in this database. All other analyzes were performed using the Minitab® 18 software (Minitab Inc., State College, PA, USA). For all analyses, significance was declared at P ≤ 0.05.

## Results

In total, 655 metabolites were identified. Metabolites were grouped into nine categories: Amino acids (n = 199), peptides (n = 27), carbohydrates (n = 29), energy metabolism (metabolites of the TCA cycle and oxidative phosphorylation, n = 12), lipids (n = 236), nucleotides (n = 46), cofactors and vitamins (n = 27), and xenobiotics (n = 79).

### Influenced metabolites by treatments and age

**Fig 2:**
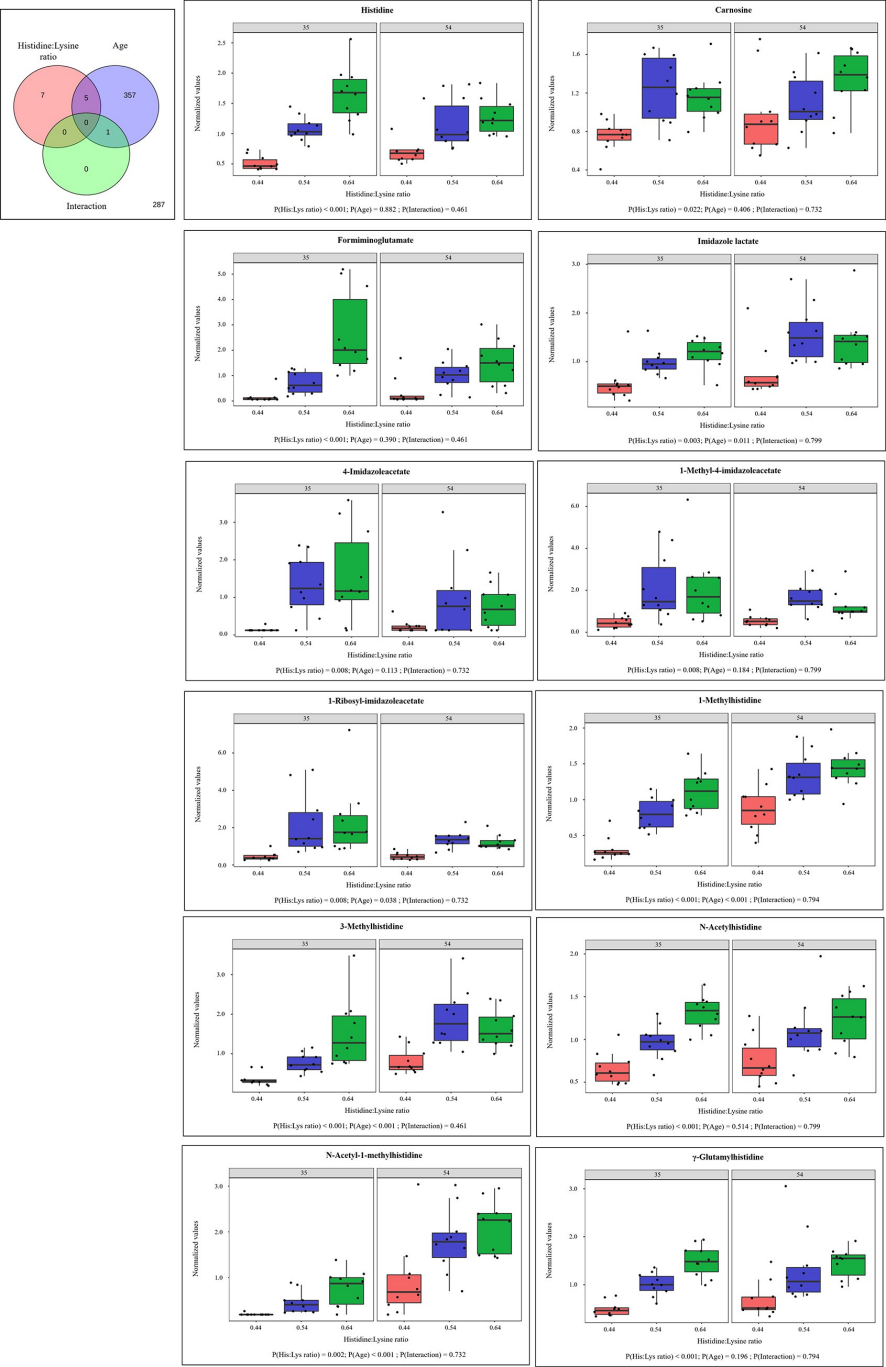
Two-factor analysis by using standardized ileal digestible histidine:lysine ratio in feed and slaughter age as factor. (A) Venn diagram of dietary standardized ileal digestible histidine:lysine ratio and slaughter age as parameters. Differences in metabolite concentrations were analyzed using a 2-way ANOVA. Metabolites were separated according to the statistically significant effects of the parameter’s standardized ileal digestible histidine:lysine ratio in the diet (0.44, 0.54, and 0.64, respectively), age at slaughter (35 d and 54 d, respectively), and the interaction of both. Significance was indicated at P ≤ 0.05; (B) Boxplots of the significantly different metabolites by histidine:lysine ratio; One-way ANOVA results were generated using Minitab18®; Abbreviations: His:Lys, standardized ileal digestible histidine:lysine ratio.

**Fig 3:**
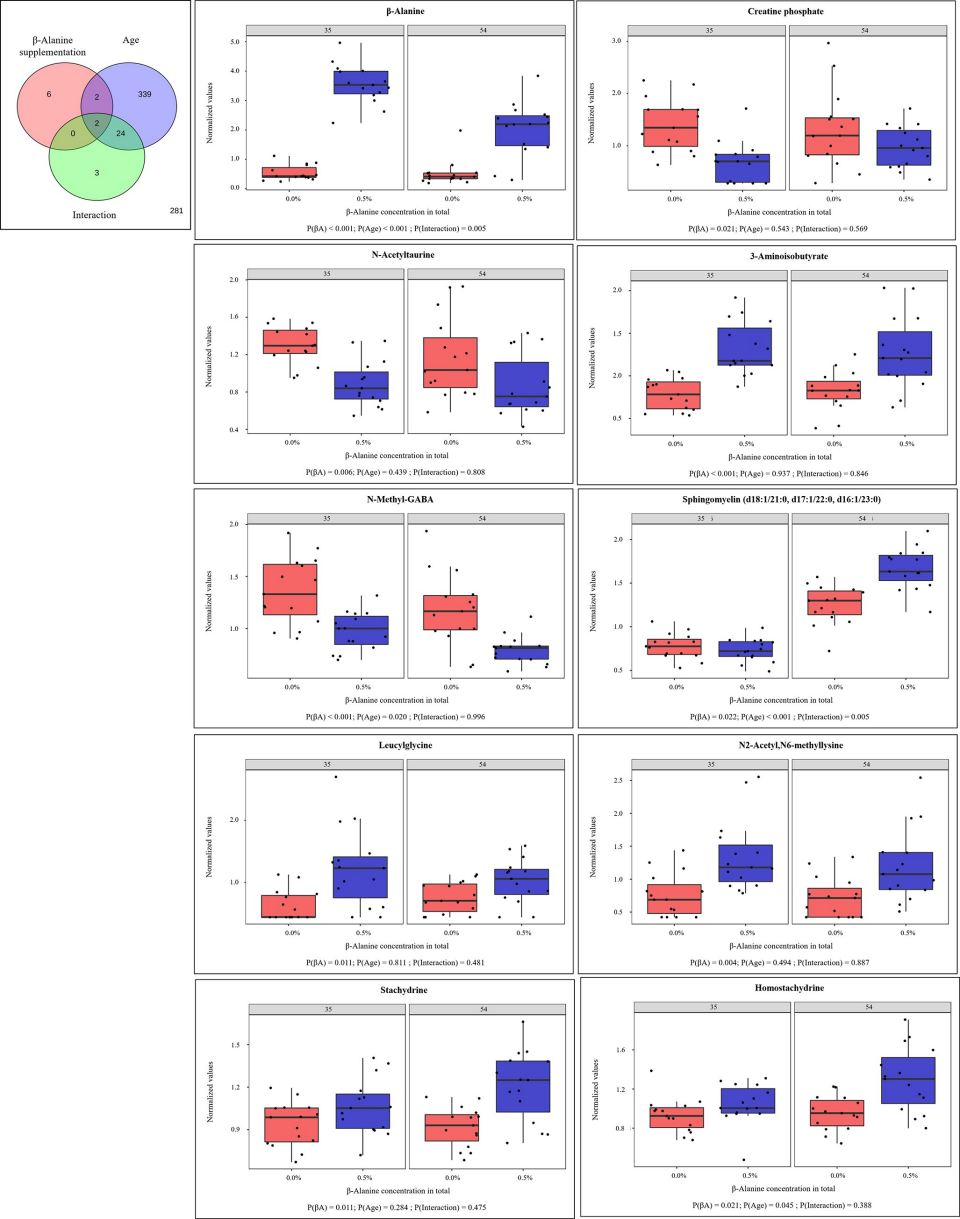
Two-factor analysis by using β-alanine supplementation in feed and slaughter age as factor. (A) Venn diagram of dietary β-alanine supplementation and slaughter age as parameters. Differences in metabolite concentrations were analyzed using a 2-way ANOVA. Metabolites were separated according to the statistically significant effects of the parameters β-alanine supplementation in feed (0% and 0.5%), age at slaughter (35 d and 54 d of age), and the interaction of both. Significance was indicated at P ≤ 0.05; (B) Boxplots of the significantly different metabolites by β-alanine supplementation. Abbreviations: βA, β-alanine.

To analyze possible effects of His:Lys ratio, βA supplementation, and slaughter age, as well as interactions of these factors a two-factor analysis was performed. In a two-factor analysis, no interactions were found between the His:Lys ratio and the βA-supplements in both slaughter ages. Therefore, both parameters were analyzed separately combined with slaughter age as factor. When the His:Lys ratio or the βA-supplements were analyzed together with the slaughter age, the most significantly different metabolites were detected for slaughter age (Figs 2A and 3A). The 12 metabolites that were significantly different for the His:Lys ratios are shown in Fig 2B. All these metabolites belong to His metabolism, including the dipeptides γ-glutamylhistidine and carnosine. The same metabolites differed significantly when the His:Lys ratio and βA additions at both slaughter ages were used as factors for two-factor analysis, except for *N*-acetyl-1methylhistidine, which was affected only by two-factor analysis of the His:Lys ratio and slaughter age. Four metabolites of His metabolism were influenced only by slaughter age: 1-methylhistamine (P = 0.023), *trans*-urocanate (P = 0.003), 1-methyl-5-imidazoleacetate (P = 0.022), and N-acetyl-3-methylhistidine (P <0.001). Two-factor analysis of βA supplementation and slaughter age identified 10 metabolites that were significantly affected by βA supplementation (Fig 3B). The metabolite βA showed differences for βA supplementation, slaughter age, and the interaction of both in blood plasma. The metabolites N-methyl-GABA, N-acetyltaurine, and creatine phosphate were decreased by 0.5% βA supplementation in broiler diets. The other affected metabolites found at higher concentrations by feeding 0.5% βA were 3-aminoisobutyrate, N2-acetyl-N6-methyllysine, leucylglycine, sphingomyelin d18:1/21:0, d17:1/22:0, d16:1/23:0, and the xenobiotics stachydrine and homostachydrine. The same metabolites differed significantly with βA-supplementation when using His:Lys ratio and βA supplementation as factors for two-factor analysis, except for sphingomyelin d18:1/21:0, d17:1/22:0, d16:1/23:0. Slaughter age had the greatest effect on metabolite concentration. Of all metabolites, 55.6% (n = 364) and 56.0% (n = 367) showed significant differences in the analysis of His:Lys and βA supplementation, respectively, in a two-factor analysis with age as the second factor.

### Effected metabolic pathways by treatments and age

**Fig 4:**
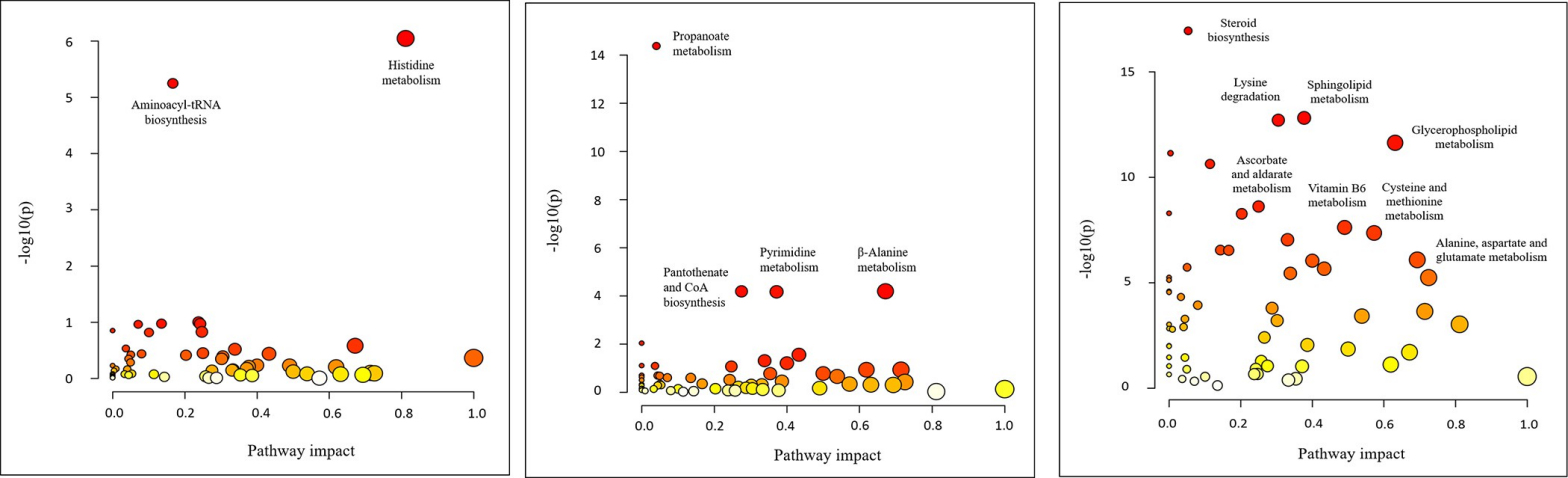
Summary of pathway analysis by using MetaboAnalyst 5.0. The color of the given dots are based on the P-value and the radius is based on the pathway impact value. (A) Pathway analysis by separating the data by standardized ileal digestible histidine:lysine ratios in the feed (0.44; 0.54 and 0.64, repectively); (B) Pathway analysis by separating the data by β-alanine supplementation in the feed (0% and 0.5%, repectively); (C) Pathway analysis by separating the data by slaughter ages (35 d and 54 d, respectively).

A metabolic pathway analysis was performed to analyze the effects of dietary His:Lys ratio, βA supplementation, and slaughter age on metabolic pathways. Analysis of the effects of His:Lys ratio in the diet and βA supplementation showed a small effect for these two factors. Variation of His in the diet mainly affected His metabolism. In addition, aminoacyl-tRNA biosynthesis was affected, but with less influence on the metabolic pathway (Fig 4A). Supplementation of βA affected βA-alanine metabolism, pyrimidine metabolism, pantothenate-CoA biosynthesis, arginine and proline metabolism, and glyoxylate and dicarboxylate metabolism. Propanoate metabolism was also affected, but with less impact on the metabolic pathway (Fig 4B). Unlike the His:Lys ratio in the diet and βA supplementation, where only a few metabolic pathways were affected, many metabolic pathways were affected by the age of the birds (Fig 4C). The metabolic pathways affected included amino acid, nucleotide, lipid, cofactor, and carbohydrate metabolic pathways. In addition, His and βA metabolism were also strongly affected by the age of the birds. The metabolic pathways affected and the exact values of the effects, as well as the statistical relevance of each, are shown in Table 1.

**Table 1:** Impacted pathways by the different parameters as analyzed by pathway analysis.

Parameter	Metabolic pathway	Impact value	Probability
**His:Lys ratio** ^**a**^	Histidine metabolism	0.81	<0.001
	Aminoacyl-tRNA biosynthesis	0.17	<0.001
**βA supplementation** ^**b**^	β-Alanine metabolism	0.67	<0.001
	Arginine and proline metabolism	0.43	0.028
	Pyrimidine metabolism	0.37	<0.001
	Glyoxylate and dicarboxylate metabolism	0.34	0.049
	Pantothenate-CoA biosynthesis	0.28	<0.001
	Propanoate metabolism	0.04	<0.001
**Slaughter age**	Histidine metabolism	0.81	0.001
	Glycine/serine and threonine metabolism	0.72	<0.001
	Taurine and hypotaurine metabolism	0.71	<0.001
	Alanine/aspartate and glutamate metabolism	0.69	<0.001
	β-Alanine metabolism	0.67	0.020
	Glycerophospholipid metabolism	0.63	<0.001
	Cysteine and methionine metabolism	0.57	<0.001
	D-Glutamine and D-glutamate metabolism	0.50	0.014
	Vitamin B6 metabolism	0.49	<0.001
	Sphingolipid metabolism	0.38	<0.001
	Arachidonic acid metabolism	0.33	<0.001
	Lysine degradation	0.31	<0.001
	Ascorbate and aldarate metabolism	0.25	<0.001
	Pentose and glucuronate conversions	0.20	<0.001
	Terpenoid backbone biosynthesis	0.11	<0.001
	Steroid biosynthesis	0.05	<0.001

Slaughter age is repharing to the ages 35 d and 54 d, respectively

^a^His:Lys ratio: standardized ileal digestible histidine:lysine ratios in the feed (0.44; 0.54 and 0.64, repectively)

^b^βA supplementation: β-alanine supplementation in the feed (0% and 0.5%, repectively)

### Effects of the different feeding groups on histidine metabolism

The group-wise analysis, as shown in the statistical heat map (Table 2), is intended to provide an overview of the differences in the His pathway due to the different feeding groups and slaughter ages for each group. Few metabolites of His metabolism had higher mean values in 54 d old birds than in 35 d old birds. The concentration of His in plasma was higher in the CON, HIS1, and BA groups at 54 d of age than in younger birds. Most changes between slaughter ages were observed in the methylated and acetylated forms of His. In addition, the metabolite N-acetyl-1-methylhistidine was affected by all age-related comparisons. The metabolite *trans*-urocante, which is related to the catabolism of His, was more concentrated in all groups supplemented with βA at 54 d of age. In contrast to the comparison of feeding groups, anserine was affected by slaughter age in HIS1, HIS2, BA_CON, and BA_HIS1. The concentration of βA was also age-dependent and decreased in the BA_CON and BA_HIS1 groups at 54 d of age. Many metabolites had higher concentrations when the experimental groups were compared with CON at both ages. As with the age comparison, many changes in methylated and acetylated forms of His were noted, but catabolism of His and histamine metabolism were also strongly affected in all groups that received a higher ratio of His:Lys compared with the control. The BA_CON group showed the least changes in the concentration of metabolites. After 35 d, only βA showed higher concentrations compared to CON. In addition, βA was less concentrated in HIS1 and HIS2 after 35 d, as well as in HIS1 after 54 d. The dipeptide carnosine showed no effect when comparing BA_CON and CON at both ages. The metabolites 4-Imidazole-5-propionate, glutamate, anserine, and histamine were not affected by the comparison of feeding groups at both ages. Relating the variation in the metabolome to variation in performance, as indicated by Lackner et al. 2021 [23], would have been interesting, but was not possible due to the small number of birds per group and the pen-wise recording of performance.

**Table 2:** Statistical heat map of the histidine pathway and related metabolites, as well as inflammation marker and standards.

Metabolite	Slaughter age effects ^a^						Feeding group effects at 35 d^b^					Feeding group effects at 54 d^c^				
	CON^d^ 54 d/35d	HIS1^e^ 54 d/35 d	HIS2^f^ 54 d/35 d	BA_CON^g^ 54 d/35 d	BA_HIS1^h^ 54 d/35 d	BA_HIS2^i^ 54 d/35 d	HIS1/CON	HIS2/CON	BA_CON/ CON	BA_HIS1/CON	BA_HIS2/CON	HIS1/CON	HIS2/CON	BA_CON/ CON	BA_HIS1/CON	BA_HIS2/CON
**Histidine**	1.30	0.98	0.78	1.70	1.15	0.76	2.16	3.37	1.10	2.28	3.42	1.63	2.04	1.44	2.02	2.01
**1-Methylhistidine**	1.94	1.58	1.17	3.94	1.73	1.40	2.22	3.20	0.79	2.44	3.27	1.80	1.93	1.60	2.18	2.36
**3-Methylhistidine**	1.70	2.26	1.21	2.93	2.68	0.92	2.00	4.28	0.86	1.99	3.61	2.66	3.05	1.48	3.14	1.96
**N-Acetylhistidine**	1.15	1.20	0.95	1.16	1.09	0.93	1.53	2.02	1.18	1.68	2.33	1.60	1.66	1.18	1.60	1.89
**N-Acetyl-3-methylhistidine**	1.00	2.97	2.41	1.36	3.15	2.24	1.00	1.59	1.00	1.20	1.00	2.97	3.83	1.36	3.79	2.24
**N-Acetyl-1-methylhistidine**	2.17	4.00	2.77	7.66	3.96	2.76	2.07	3.24	0.92	2.41	4.32	3.80	4.13	3.26	4.39	5.48
***trans*-Urocanate**	0.86	5.40	1.59	4.98	3.72	4.16	1.79	5.21	0.70	1.29	3.79	11.25	9.62	4.04	5.56	18.3
**4-Imidazole-5-propionate**	1.56	4.12	1.30	1.38	3.25	1.73	0.52	0.92	0.67	0.63	0.64	1.37	0.76	0.59	1.31	0.71
**Formiminoglutamate**	0.34	1.59	0.60	6.89	1.21	0.53	2.94	12.19	0.36	3.1	9.93	13.80	21.74	7.40	11.11	15.67
**Glutamate**	0.8	0.96	0.95	0.77	0.74	0.95	0.94	0.94	0.98	1.2	0.86	1.13	1.11	0.95	1.11	1.02
**Imidazole lactate**	0.88	1.75	0.89	2.16	1.38	1.55	1.65	1.78	0.65	1.28	1.69	3.30	1.81	1.61	2.03	2.99
**Carnosine**	1.09	0.91	1.31	1.43	0.86	1.03	1.50	1.37	1.09	1.92	1.83	1.25	1.65	1.43	1.52	1.75
**Acetylcarnosine**	1.10	0.79	1.11	1.80	0.98	1.16	1.93	1.74	1.04	1.66	2.04	1.38	1.76	1.71	1.48	2.16
**Anserine**	1.36	1.43	1.55	1.77	1.63	1.23	0.99	0.97	0.75	0.96	1.00	1.04	1.11	0.97	1.15	0.90
**Histamine**	1.00	0.45	0.61	0.62	0.87	0.55	2.11	1.39	1.24	2.2	1.39	0.95	0.85	0.77	1.90	0.77
**1-Methylhistamine**	0.97	0.25	0.64	0.82	0.34	0.84	12.86	4.63	1.72	7.23	4.95	3.35	3.04	1.45	2.53	4.26
**1-Methyl-4-imidazoleacetate**	1.24	0.58	0.74	1.08	1.05	0.51	5.66	3.80	1.12	3.96	5.72	2.66	2.26	0.97	3.36	2.38
**1Mmethyl-5-imidazoleacetate**	0.80	1.62	0.89	1.2	2.87	1.34	1.04	1.92	0.91	0.86	1.20	2.11	2.11	1.36	3.08	2.01
**1-Ribosyl-imidazole-4-acetate**	0.93	0.56	0.66	1.18	0.71	0.43	5.48	3.82	0.87	3.72	6.09	3.27	2.69	1.11	2.84	2.79
**Imidazole-4-acetate**	1.42	0.68	0.43	1.71	0.80	0.51	11.94	11.03	1.27	9.6	14.31	5.75	3.37	1.53	5.39	5.10
**Acetylhistamine**	1.66	0.80	0.55	1.34	1.34	0.55	5.20	4.32	1.44	2.63	4.22	2.52	1.44	1.17	2.12	1.41
**β-Alanine**	0.67	0.75	1.95	0.58	0.43	0.72	0.48	0.40	4.72	4.01	3.69	0.53	1.17	4.09	2.55	3.97
***gamma*-Glutamylhistidine**	1.06	1.14	0.95	1.66	1.48	0.99	2.00	3.36	1.16	2.4	3.17	2.14	3.01	1.81	3.35	2.96
**Creatinine**	1.14	0.98	1.07	1.24	0.99	1.03	1.15	0.96	0.85	0.99	0.94	0.99	0.9	0.92	0.86	0.85
**Kynurenine**	0.93	0.99	0.91	1.27	1.37	1.67	1.03	0.87	0.90	0.74	0.76	1.10	0.85	1.23	1.10	1.36
**Tryptophan**	0.97	0.89	0.87	0.89	0.97	0.96	0.97	1.01	0.99	0.99	0.95	0.88	0.91	0.91	0.99	0.94

For each metabolite, an average fold change (mean ratio difference between the two groups) was calculated based on age at slaughter and the effects of feeding group at both slaughter ages (at d 35 and 54, respectively). For a given comparison, the red and green colors indicate the relative significance increased and decreased concentrations, respectively, for each metabolite. Normalized and rescaled data were used for the analysis. Significance was declared by P ≤ 0.05.

^a^The effects on the given metabolites by slaughter age (35d and 54 d) were analyzed for each feeding group

^b^The effects on the given metabolites at the slaughter age of 35 d were analyzed for each supplemented feeding group compared to the control group

^c^The effects on the given metabolites at the slaughter age of 54 d were analyzed for each supplemented feeding group compared to the control group

^d^CON: group fed with a standardized ileal digestible histidine:lysine ratio of 0.44

^e^HIS1: group fed with a standardized ileal digestible histidine:lysine ratio of 0.54

^f^HIS2: group fed with a standardized ileal digestible histidine:lysine ratio of 0.64

^g^BA_CON: group fed with a standardized ileal digestible histidine:lysine ratio of 0.44 + supplemented of 0.5% β-alanine

^h^BA_HIS1: group fed with a standardized ileal digestible histidine:lysine ratio of 0.54 + supplementation of 0.5% β-alanine

^i^BA_HIS2: group fed with a standardized ileal digestible histidine:lysine ratio of 0.64 + supplementation of 0.5% β-alanine

To analyze protein turnover and inflammatory conditions, some additional analyzes were performed. Protein turnover was analyzed by the 3-methylhistidine:creatinine ratio. The 3-methylhistidine:creatinine ratio increased with dietary His:Lys ratio (P < 0.001, His:Lys 0.44 = 0.63, His:Lys 0.54 = 1.07, His:Lys 0.64 = 1.51) and with βA supplementation (P = 0.021, 0% βA = 0.97, 0.5% βA = 1.17) using a linear model for ANOVA. Age had no significant effect on the ratio of 3-methylhistidine to creatinine. The ratio of kynurenine to tryptophan was used as a marker for inflammation. In a linear model for ANOVA, the ratio of kynurenine to tryptophan, as a marker of inflammation [28], was influenced only by age (P < 0.001, 35 d = 0.92, 54 d = 1.16), but not by experimental His or βA supplementation. In addition, interactions were found between age and βA supplementation (P = 0.001) and His:Lys ratio and βA supplementation (P = 0.004). The results for the group-wise analysis of these metabolites were additionally reported in Table 2.

## Discussion

Two-factor analysis of the His:Lys ratio and slaughter age, as well as metabolic pathway analysis for the His:Lys ratio, showed that the different ratios mainly affected His metabolism. Several metabolites of His metabolism were more abundant in the groups fed with higher His:Lys ratios, especially formiminoglutamate and imidazole lactate. The predominant metabolites of His catabolism are shown in the heat map (Table 2); formiminoglutamate which is the direct link between His and glutamate metabolism, was also found significant in the two-factor analysis (Fig 2B). It appears that oversupplied His is mainly catabolized towards glutamate and the citric acid cycle, as also stated for mammals [6]. Nevertheless, heat map analysis of glutamate and other metabolites did not reveal higher levels than those found at CON. Moreover, urocanate and 4-imidazole-5-propionate as precursors of formiminoglutamate were also not affected by the His:Lys ratios used, as analyzed by two-factor analysis (Fig 2B). This could indicate a fast-catalytic mechanism for formiminoglutamate and a slow turnover for glutamate. The greater concentration of formiminoglutamate could be the result of His loading by feeding His:Lys ratios of 0.54 and 0.64. A high concentration of formiminoglutamate in mammalian urine is also known to be a marker of folate or cobalamin deficiency [24, 25]. This is caused by the use of tetrahydrofolate (**THF**) by the enzyme formimidoyltransferase-cyclodeaminase. This enzyme transfers the formimine group to THF, resulting in glutamate as end product. It can be speculated that higher exposure to His, and thus higher turnover towards glutamate, results in lower THF levels and an increase in formiminoglutamate in the metabolism of broiler chickens. The cofactor cobalamin is also coupled to folate and His metabolism via the enzyme methionine synthase. This enzyme uses homocysteine and N5-methyl-THF to form methionine and THF. Therefore, a high concentration of formiminoglutamate could also be a consequence of cobalamin deficiency [29]. However, other THF-dependent metabolic pathways such as serine/glycine metabolism, methionine metabolism, or tryptophan metabolism showed no changes. The second intermediate of His catabolism detected when feeding a higher His:Lys ratio was imidazole lactate, which is formed from the previous metabolite imidazole acetate. It was pointed out that the minor catabolism of His to imidazole acetate occurs at high His concentrations and imidazole acetate can be further catabolized to imidazole lactate [3]. In addition, the intermediates imidazole-4-acetate and 1-methyl-4-imidazole acetate, the final metabolites of the two ways of histamine catabolism, were detected at higher concentrations when fed with higher ratios of His:Lys. The concentration of the His-related dipeptide carnosine was greater at the higher ratio of His:Lys, whereas no differences were detected for the methylated form anserine. However, these dipeptides are naturally degraded by carnosinase activity in blood plasma, whereas synthesis occurs in tissues such as skeletal muscle and brain [9, 30] where high concentrations of these two dipeptides have previously been reported following dietary supplementation with His [13, 23, 31]. In the study of Kai et al. 2015 [13] it was reported that no dipeptides were detectable in the blood serum of broiler chickens when fed a high-His diet, which is in line with our results. Indeed, in another analysis of the same blood samples, plus 5 additional samples per bird of the same study, carnosine was significantly higher in plasma of birds fed a higher ratio of His:Lys at both ages [23]. In this analysis, anserine was also found in higher concentrations in plasma of birds fed a His:Lys ratio of 0.64 compared to 0.44 at 35 days of age [23]. The two-factor analysis shows that the metabolism of His depends not only on the His concentration in the diet but also on the age of the bird. The molecules involved were: *trans*-urocanate, 1-methyl-5-imidazoleacetate, 1-methylhistamine, and N-acetyl-3-methylhistidine. The fact that almost exclusively metabolites of His metabolism were affected by differences in the His:Lys ratio in the diet also suggests that such a metabolic study of the amino acid His is well suited to investigate the effects of His on the broiler chickens. In addition, it has to be mentioned that the metabolic pathway analysis revealed that His metabolism was affected by age.

Metabolomics analysis of βA supplementation revealed affected metabolites from several metabolic pathways, not all of which can be directly linked to βA metabolism. As expected, βA was also more concentrated in plasma samples from broilers fed 0.5% βA. Moreover, βA showed high impact on its associated metabolic pathways pyrimidine metabolism and pantothenate and CoA biosynthesis. The catabolism of βA generates malonate semialdehyde and acetyl-CoA. The first step is catalyzed by 4-aminobutyrate aminotransferase to form malonate semialdehyde. This enzyme is also known to catalyze the reaction of methylmalonate semialdehyde to 3-aminoisobutyrate [32]. It seems possible that the upregulation of this enzyme due to the high βA content in broiler feed could be the reason for the higher concentration of 3-aminoisobutyrate. All other metabolites involved, with the exception of the xenobiotics stachydrine and homostachydrine, could be generally related to βA metabolism but are not directly known to be part of this metabolism. Stachydrine and homostachydrine are mainly plant-derived compounds that are strongly associated with fruits [33]. Therefore, the higher concentrations of these metabolites could be a consequence of diet, but these metabolites were only detected significantly different in plasma samples from birds fed high levels of βA, which could indicate an interaction between βA supplementation and stachydrine metabolism in birds. The main reason for using βA supplementation in this study was to determine the effects of this metabolite on the metabolism of His, in which it is involved through the formation of the His-related dipeptides carnosine and anserine, which are found in high concentrations in chicken skeletal muscle [9]. The heat map analysis showed that βA-supplementation had no effect on the plasma concentrations of these dipeptides. This was also shown for both dipeptides in skeletal muscle tissue in the companion study [23]. Nevertheless, some metabolites of His metabolism after 54 d of age were affected by βA supplementation in the BA_CON group, but to a much lesser extent than in the other feeding groups. These results highlight previous findings that βA supplementation had no effect on carnosine and anserine concentrations in broiler chickens, even at higher His levels in the broiler diet, and that His therefore appears to be the limiting factor for dipeptide formation, which was also reported in a previous study [23]. It should be noted that both dipeptides are degraded in blood plasma by carnosinase activity in blood serum and their concentrations are low [30, 34]. A decrease of carnosine in blood plasma after βA supplementation was previously described [34]. The authors of this study also reported no changes of the anserine concentration in blood plasma when βA or His alone or in combination were added to the broiler feed.

Age showed the highest effect on the metabolism of birds; lipid, amino acid, vitamin, and carbohydrate metabolisms were mainly affected. The metabolism of His was also affected by the age of the birds, as well as βA metabolism, which was lowered in older birds. The metabolite βA can be formed by various metabolic pathways. Among them, decarboxylation of aspartate leads to direct formation of βA. This metabolic pathway was also influenced by age in the metabolic pathway analysis. When metabolites that affected this analysis were analyzed, aspartate was found to be lower in older birds. In addition, concentrations of spermidine and spermine, which are also precursors of βA, were decreased at 54 d. Interestingly, age but not the His:Lys ratio in feed or a supplementation of βA was significant for the dipeptide anserine. In breast muscle tissue, anserine was also affected by age [23]. The age-related increase of anserine in blood plasma may indicate that methylation of carnosine in muscle is a slow process.

The two-factor analysis and heat map showed an effect on 3-methylhistidine by age and His:Lys ratio. In skeletal muscle, His is methylated and used as a building block for actin and myosin. When myofibrillar protein is degraded, 3-methylhistidine is released but cannot be further used for protein synthesis and is thus renally excreted. Approximately 84% of 3-methylhistidine is expected to be derived from broiler chicken muscle tissue, with actin being the major source [35]. Therefore, the metabolite in blood and urine can be used as a marker of muscle protein degradation and turnover [36–38]. In this study, the concentration of 3-methylhistidine increased with slaughter age in the CON, HIS1, BA_CON, and BA_HIS1 groups and all feeding groups with an increased His:Lys ratio of 0.54 and 0.64 (Table 2). By using creatinine as a standard that depends on muscle mass and age, the His:creatinine ratio can be used as a great indicator of muscle protein turnover by normalizing the effects of age and muscle growth. The results for the analyzed His:creatinine ratio possibly indicates a high turnover of muscle protein with high dietary His supply. Moreover, it could indicate that the birds used for sampling were affected of muscle defects such as white striping, woody breast, or spaghetti meat. Unfortunately, myopathies could not be assessed for technical reasons in our study, but a myodegeneration in affected muscles is described in previous literature [39]. Inflammation also occurs in muscle tissue affected by breast myopathies in chicken [39]. The ratio of kynurenine to tryptophan can be used as a marker of inflammation because of its dependence on the activity of indolamine-2,3-dioxygenase, an enzyme activated by cytokines [28]. The analyzed increased ratio for this inflammatory marker in older birds may indicate a higher incidence of breast myopathies [39]. However, it should be noted that the plasma concentration of these metabolites also depends on other metabolic factors and inflammatory processes, or the overall regulation of tryptophan metabolism [28].

In all interpretations of the results, it must be mentioned that this study had some limitations. For this metabolic study, only five samples were collected for each feeding group, and the analyzed values of these samples were pooled for statistical analysis according to the factors His:Lys ratio, βA intake, and slaughter age. It should also be noted that the missing values from the original analytical methods were annotated with the observed minimum value of the detected molecules after rescaling, which could affect some analyzes.

In conclusion, a higher supplementation of His or βA showed less effect on the metabolism of broiler chickens. The His:Lys ratio showed a direct influence on the targeted His metabolism, while the supplementation of βA affected a few metabolites of different metabolic pathways. A higher His:Lys ratio led to higher concentrations of formiminoglutamate in blood plasma, which could be an indicator for a folate deficiency. The dipeptide carnosine, which is built by His and βA, was only increased with a higher His:Lys ratio in broiler feed, whereas the methylated form anserine was increased by age. In general, age had the greatest influence on the metabolism of the birds.

## Supporting information

S1 File Rescaled and imputed raw data as used for analysis. The original values were normalized in terms of raw area counts. All data were rescaled by the median of all 60 samples for a given molecule. Missing values were imputed with the observed minimum value for each detected metabolite.
(XLSX)
